# Long-term outcomes of endoscopic radiofrequency ablation for pyriform sinus fistula in children and risk factors for transient vocal cord paralysis

**DOI:** 10.3389/fped.2026.1774182

**Published:** 2026-03-06

**Authors:** Huihui Niu, Bingliang Li, Xiangbin Chai, Wenjuan Li

**Affiliations:** 1 Department of Otolaryngology, Shanxi Children's Hospital (Shanxi Maternal and Child Health Hospital), Taiyuan, China; 2 Shanxi Medical University, Taiyuan, China; 3 Department of Neonatal Surgery, Shanxi Children's Hospital (Shanxi Maternal and Child Health Hospital), Taiyuan, China; 4 First Hospital of Shanxi Medical University, Taiyuan, China

**Keywords:** radiofrequency ablation, minimally invasive surgery, pyriform sinus fistula, risk factors, vocal cord paralysis, children

## Abstract

**Objectives:**

To evaluate the long-term efficacy and safety of endoscopic low-temperature plasma radiofrequency ablation (coblation) for the treatment of pyriform sinus fistula (PSF) in children, and to identify risk factors associated with postoperative transient vocal cord paralysis.

**Methods:**

A retrospective cohort study was conducted at a single tertiary pediatric center. Children with pyriform sinus fistula who underwent endoscopic coblation were consecutively enrolled. Demographic characteristics, perioperative variables, postoperative complications, and follow-up outcomes were collected. Univariate and multivariate logistic regression analyses were performed to identify independent risk factors for postoperative transient vocal cord paralysis.

**Results:**

Endoscopic coblation was successfully performed in all patients. During follow-up, the majority of children experienced no recurrent cervical infection or fistula recurrence. Endoscopic examinations confirmed complete closure of the internal opening in the pyriform sinus, indicating favorable long-term outcomes and good procedural reproducibility. Transient vocal cord paralysis occurred in some patients but resolved completely in all affected cases, with no permanent nerve injury. Multivariate logistic regression analysis identified younger age and elevated preoperative white blood cell counts as independent risk factors for transient vocal cord paralysis.

**Conclusions:**

Endoscopic coblation is a safe, effective, and repeatable minimally invasive treatment for pediatric PSF, with low recurrence rates and stable long-term outcomes. Postoperative transient vocal cord paralysis is a relatively common but reversible complication, closely associated with younger age and elevated preoperative inflammatory status. These risk factors suggest that intraoperative strategies, such as adjusting ablation depth in very young children and ensuring adequate thermal dissipation, may help mitigate this risk.

## 1 Introduction

Pyriform sinus fistula (PSF) is a rare congenital branchial anomaly caused by abnormal development of the third or fourth pharyngeal pouch during embryogenesis. Approximately 80% of cases are diagnosed in childhood (1, 2). The lesion predominantly affects the left side. Its characteristic pathological feature is an internal opening at the base of the pyriform sinus that communicates with the pharyngeal cavity. Through this tract, pathogens can repeatedly enter the cervical tissues, leading to persistent or recurrent infections. PSF is therefore an important cause of cervical infection and acute suppurative thyroiditis in children (3, 4).Because the fistulous tract is often concealed and clinical manifestations are nonspecific, PSF is frequently misdiagnosed or overlooked. Affected children often undergo repeated courses of antibiotic therapy or even cervical incision and drainage. These delays not only postpone definitive treatment but also increase surgical complexity and the risk of postoperative complications (5).

Traditional open excision of the fistulous tract can eradicate the lesion but is associated with substantial surgical trauma and a relatively high complication rate (6). In recent years, minimally invasive endoscopic strategies focusing on closure of the internal opening have become the preferred treatment for pediatric PSF. Among these techniques, low-temperature plasma radiofrequency ablation (coblation) has demonstrated favorable outcomes in interrupting the infectious pathway and reducing recurrence rates (7). However, postoperative vocal cord paralysis remains a relatively common complication of this procedure, and its associated risk factors have not been systematically evaluated. Therefore, this retrospective study analyzed pediatric patients with PSF who underwent endoscopic low-temperature plasma radiofrequency ablation at our institution. We aimed to assess the long-term efficacy and safety of this technique and to identify risk factors for postoperative transient vocal cord paralysis, with the goal of providing evidence to support perioperative risk assessment and optimization of surgical strategies.

## 2 Materials and methods

### 2.1 Patients

This retrospective study included pediatric patients with PSF who underwent endoscopic radiofrequency ablation of the internal opening at our institution. All patients presented with symptoms related to recurrent cervical infection or acute suppurative thyroiditis.

Preoperative evaluation routinely included cervical ultrasonography, contrast-enhanced computed tomography (CT), and flexible laryngoscopy to confirm the diagnosis and assess lesion characteristics. Imaging studies were primarily used to evaluate the extent of infection, the presence of abscess formation, and the course of the fistulous tract. The presence of gas within the tract or infected area on contrast-enhanced CT was considered a characteristic finding suggestive of PSF. Endoscopic laryngoscopic examination, usually performed during the non-acute phase, allowed direct visualization of the internal opening in the pyriform sinus and served as the key diagnostic modality.

All patients in the acute infection stage first received standardized anti-infective treatment. Endoscopic radiofrequency ablation was performed only after the patients were clinically stable, local infection was well controlled, and inflammatory markers had improved.

### 2.2 Data collection

Clinical data were collected by reviewing electronic medical records and follow-up documentation. Baseline variables included sex, age, body mass index (BMI), initial presenting symptoms, interval from symptom onset to surgery, number of preoperative infection episodes, preoperative laboratory parameters—white blood cell count (WBC), C-reactive protein (CRP), and procalcitonin (PCT) and imaging findings.Perioperative data included surgical technique, radiofrequency ablation power settings, whether concomitant cervical incision and drainage was performed, postoperative length of hospital stay, and perioperative complications.

Postoperative follow-up was conducted through a combination of outpatient visits and telephone interviews. Follow-up assessments focused on recurrence of cervical infection or neck masses, changes in voice, and swallowing and respiratory function. All surgery-related complications were recorded. Patients with suspected recurrence or persistent symptoms underwent further imaging or laryngoscopic evaluation for confirmation. Transient vocal cord paralysis was defined as postoperative hoarseness or phonation abnormality that completely resolved during follow-up, without permanent vocal cord mobility impairment. Permanent vocal cord paralysis was defined as persistent hoarseness with no recovery during follow-up. Vocal cord function was assessed based on clinical symptoms, laryngoscopic findings, and follow-up observations. Laryngeal electromyography was not routinely performed because all cases of vocal cord paralysis were mild and completely resolved without evidence of persistent or progressive nerve dysfunction. For pediatric patients who did not exhibit postoperative hoarseness, routine laryngoscopy was not conducted to evaluate vocal cord function. This approach was taken because postoperative hoarseness in this setting is generally attributable to transient thermal stimulation rather than to structural nerve injury. It also aimed to avoid unnecessary procedural discomfort, reduce potential infection risks, and lessen the financial burden on children and their families.

### 2.3 Surgical procedure

Patients in the acute phase underwent surgery after standardized anti-infective therapy and, when necessary, cervical incision and drainage. Surgery was performed once fever and neck pain had resolved, the infectious focus had markedly decreased, and inflammatory markers had normalized or nearly normalized. None of the patients had previously undergone open fistula excision or endoscopic cauterization of the internal opening.

All procedures were performed under general anesthesia. Patients were placed in the supine position with a shoulder roll to achieve moderate neck extension. A suspension laryngoscope was inserted transorally, and the affected pyriform sinus was fully exposed under high-definition endoscopic visualization. The esophageal inlet and adjacent mucosal folds were used as anatomical landmarks to identify the internal opening.

After identification of the internal opening, a fine catheter or suction tube was inserted into the fistulous tract to aspirate residual pus or secretions. The tract was repeatedly irrigated with diluted povidone–iodine solution and normal saline to remove infectious material and necrotic tissue as completely as possible.

**Figure 1:**
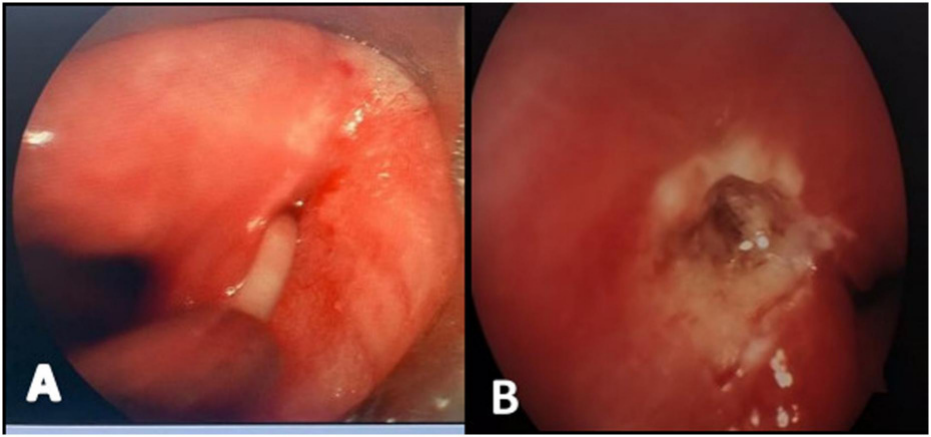
Endoscopic view under suspension laryngoscopy showing the internal opening of the pyriform sinus with purulent discharge after insertion of a plastic catheter **(A)**, and the appearance after ablation **(B)**.

A low-temperature plasma radiofrequency ablation system was then used to treat the internal opening. Power settings were standardized at 6 W for ablation mode and 2 W for coagulation mode. Centered on the internal opening, circumferential and layered ablation was performed along the proximal portion of the fistulous tract, progressing from deep to superficial layers to destroy the epithelial lining. In this study, consistent ablation parameters were employed for all patients, regardless of age. The ablation area was confined to within approximately 1 cm of the internal opening, with a target depth of 0.5 cm. This depth was chosen based on previous reports suggesting that adequate mucosal destruction (typically until the tissue turns brown) is essential for successful fistula closure while minimizing the risk of recurrence due to insufficient scarring. However, our multivariate analysis identified younger age as an independent risk factor for transient vocal cord paralysis. This likely reflects anatomical variations in younger children, such as thinner mucosal layers and more superficial nerve pathways. Therefore, although a standardized ablation depth of 0.5 cm was applied in all cases, we suggest that for children under 2 years of age, the depth may be cautiously reduced to 0.4 cm—provided that complete mucosal discoloration (browning) is achieved to ensure effective fistula closure. This modification could help minimize thermal injury to adjacent neural structures while preserving procedural efficacy. The pace of ablation was adjusted according to local tissue response to avoid excessive thermal injury (Figure 1).

Throughout the procedure, the ablation range was strictly controlled to protect surrounding critical structures, with particular attention to avoiding thermal injury to the recurrent laryngeal nerve. After ablation, the surgical field was irrigated again to confirm complete closure of the internal opening and absence of active bleeding. Patients with obvious cervical abscesses on preoperative imaging or extensive infection identified intraoperatively underwent concomitant cervical incision and drainage.

Postoperatively, intravenous antibiotics were routinely administered for approximately one week, along with supportive care. Respiratory status, swallowing function, and phonation were closely monitored.

### 2.4 Postoperative follow-up protocol and outcome assessment

In this study, a minimum follow-up of 3 months was deemed sufficient to evaluate long-term outcomes. This threshold was selected based on existing literature and our clinical experience, which indicate that most recurrences occur within the first 18 months postoperatively, with recurrence beyond this period being very unlikely in asymptomatic patients.

Postoperative follow-up consisted of scheduled clinical evaluations and assessments triggered by symptoms. All patients were assessed according to a predefined schedule at the following time points: postoperative day 1, week 1, week 2, month 1, month 2, year 1, and year 2. These evaluations focused on: (1) monitoring postoperative complications and their duration (e.g., hoarseness); (2) detecting signs of recurrence, such as neck swelling, pain, or dysphagia; and (3) documenting any need for re-intervention.

Objective examinations were performed when clinically indicated. Recurrence was defined as the reappearance of clinical symptoms suggestive of cervical infection (e.g., neck swelling, pain, or fever). Patients with suspected recurrence or persistent symptoms underwent confirmatory flexible laryngoscopy to assess internal opening closure and cervical ultrasonography to evaluate for residual or recurrent masses.

For patients who remained asymptomatic after the initial planned visits, routine clinic appointments were discontinued. Long-term follow-up was then maintained primarily through surveillance for symptom recurrence. To comprehensively ascertain the long-term status of all patients for this study, a final structured telephone interview was conducted prior to data analysis to confirm the absence of cervical infections or other related events. This approach, combining scheduled early assessments with endpoint verification, explains the reported follow-up range of 3–72 months.

### 2.5 Statistical analysis

Statistical analyses were performed using SPSS 26.0. Normality of continuous variables was assessed using the Shapiro-Wilk test. Normally distributed data are presented as mean ± standard deviation, whereas non-normally distributed data are expressed as median (interquartile range). Categorical variables are presented as counts (percentages). Between-group comparisons were performed using the independent-samples *t* test for normally distributed continuous variables, the Mann–Whitney *U* test for non-normally distributed variables, and the *χ*^2^ test or Fisher's exact test for categorical variables, as appropriate. Patients were divided into a hoarseness group and a non-hoarseness group based on the presence of postoperative transient vocal cord paralysis. Univariate logistic regression analysis was first conducted to identify potential associated factors. Variables with *P* < 0.10 in univariate analysis were entered into a multivariate logistic regression model using a stepwise approach to identify independent risk factors. Results are reported as odds ratio (OR) with 95% confidence interval (95% CI). All statistical tests were two-sided, and *P* < 0.05 was considered statistically significant.

## 3 Results

### 3.1 Comparison of baseline characteristics

A total of 36 pediatric patients with PSF were included in this study, including 21 males (58.3%) and 15 females (41.7%). The lesion predominantly involved the left side in 33 patients (91.7%), while 3 patients (8.3%) had right-sided involvement. Patient age ranged from 1.5 to 15 years, with a median age of 5 years. All patients underwent endoscopic low-temperature plasma radiofrequency ablation after control of acute infection. Preoperative evaluation routinely included cervical ultrasonography, contrast-enhanced CT, and laryngoscopic examination.

All patients presented with symptoms related to cervical infection. Cervical abscess was observed in 34 patients (94.4%), suppurative thyroiditis in 31 patients (86.1%), and fever in 14 patients (38.9%). Diagnosis was mainly established using contrast-enhanced cervical CT combined with ultrasonography in 28 patients (77.8%). 31 patients (86.1%) experienced one episode of infection prior to surgery, while 5 patients (13.9%) had more than one episode. Only one patient had a history of cervical incision and drainage. The median interval from initial symptom onset to surgery was 15 days. Concomitant cervical abscess incision and drainage was performed in 14 patients (38.9%), while 22 patients (61.1%) underwent radiofrequency ablation alone. The median maximum abscess diameter was 3.8 cm.

**Figure 2:**
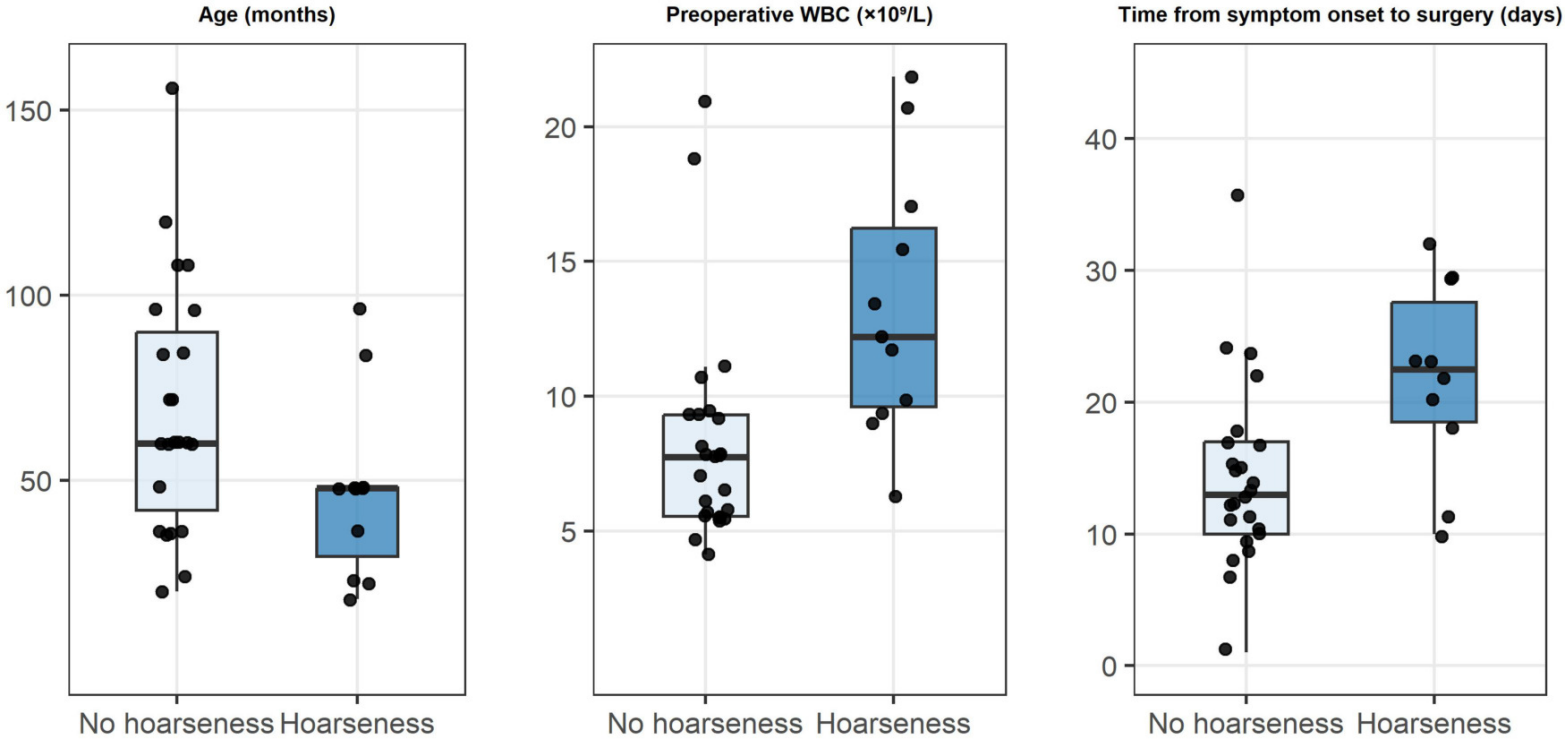
Distribution of key clinical variables between the hoarseness and non-hoarseness groups.

Postoperative transient vocal cord paralysis occurred in 11 patients (30.6%). Compared with the non-hoarseness group, patients in the hoarseness group were younger (*P* = 0.026), had a longer interval from symptom onset to surgery (*P* = 0.013), and had higher preoperative WBC levels (*P* = 0.001). No significant differences were observed between the two groups with respect to sex, BMI, lesion laterality, number of preoperative infection episodes, concomitant abscess drainage, maximum abscess diameter, preoperative CRP and PCT levels, ablation time, intraoperative blood loss, total operative time, or postoperative hospital stay (all *P* > 0.05; Table 1). Box plots (Figure 2) demonstrated a younger age distribution and higher distributions of symptom-to-surgery interval and preoperative WBC levels in the hoarseness group.

**Table 1:** Baseline characteristics and comparison between the hoarseness and non-hoarseness groups (*n* = 36).

Variable	Hoarseness group (*n* = 11)	Non-hoarseness group (*n* = 25)	Statistic	*P*
Sex			0.760	0.521
Male	6	15		
Female	5	10		
Age (months)	48 (29, 48)	60 (48, 96)	2.228	0.026
BMI (kg/m^2^)	15.21 (14.56, 17.17)	14.76 (13.92, 15.28)	1.511	0.132
Side of lesion			0.914	0.678
Left	10	23		
Right	1	2		
Interval from symptom onset to surgery (days)	23 (19, 29)	13 (10, 17)	2.459	0.013
Number of preoperative infection episodes			2.372	0.154
1	8	23		
>1	3	2		
Concomitant cervical abscess incision and drainage			0.837	0.569
Yes	4	10		
No	7	15		
Maximum abscess diameter (cm)	4.5 (3.2, 4.8)	3.5 (2.9, 4.8)	0.894	0.378
Preoperative WBC (×10⁹/L)	12.21 (9.61, 16.24)	7.78 (5.62, 9.32)	3.245	0.001
Preoperative CRP (mg/L)	7.32 (1.47, 16.73)	2.39 (0, 34.47)	0.562	0.587
Preoperative PCT (ng/mL)	0.084 (0.027, 0.167)	0 (0, 0.097)	1.471	0.183
Ablation time (s)	60 (60, 105)	60 (60, 120)	0.589	0.612
Intraoperative blood loss (mL)	1.0 (0.5, 2.5)	1.0 (0.5, 3)	0.262	0.813
Total operative time (min)	32.55 ± 4.13	36.92 ± 2.79	0.869	0.391
Length of hospital stay (days)	11.91 ± 1.39	11.24 ± 0.59	0.522	0.605

### 3.2 Univariate logistic regression analysis of transient vocal cord paralysis

Univariate logistic regression analysis showed that age (OR = 0.972, 95% CI 0.943–0.998, *P* = 0.046) and preoperative WBC level (OR = 1.268, 95% CI 1.055–1.523, *P* = 0.011) were significantly associated with postoperative transient vocal cord paralysis (Table 2). The interval from symptom onset to surgery (OR = 1.073, 95% CI 0.991–1.162, *P* = 0.084), BMI (OR = 1.472, 95% CI 0.928–2.333, *P* = 0.100), and more than one preoperative infection episode (OR = 2.049, 95% CI 0.854–4.911, *P* = 0.107) showed a trend toward association but did not reach statistical significance. Other variables, including sex, lesion laterality, concomitant abscess drainage, maximum abscess diameter, preoperative CRP and PCT levels, ablation time, intraoperative blood loss, total operative time, and length of hospital stay, were not significantly associated with transient vocal cord paralysis (all *P* > 0.05).

**Table 2:** Univariate logistic regression analysis of transient vocal cord paralysis.

Variable	*OR*	95% *CI*	*P*
Sex (male)	1.250	0.299–5.230	0.760
Age	0.972	0.943–0.998	0.046
BMI	1.472	0.928–2.333	0.100
Side of lesion (left)	1.115	0.093–14.188	0.913
Interval from symptom onset to surgery	1.073	0.991–1.162	0.084
Number of preoperative infection episodes (>1)	2.049	0.854–4.911	0.107
Concomitant cervical abscess incision and drainage (no)	1.167	0.269–5.054	0.837
Maximum abscess diameter	1.081	0.686–1.704	0.737
Preoperative WBC	1.268	1.055–1.523	0.011
Preoperative CRP	1.005	0.985–1.027	0.611
Preoperative PCT	1.467	0.018–24.962	0.787
Ablation time	0.995	0.976–1.015	0.633
Intraoperative blood loss	0.976	0.924–1.031	0.381
Total operative time	0.898	0.605–1.331	0.591
Length of hospital stay	1.058	0.681–1.300	0.594

### 3.3 Multivariate logistic regression analysis of transient vocal cord paralysis

**Figure 3:**
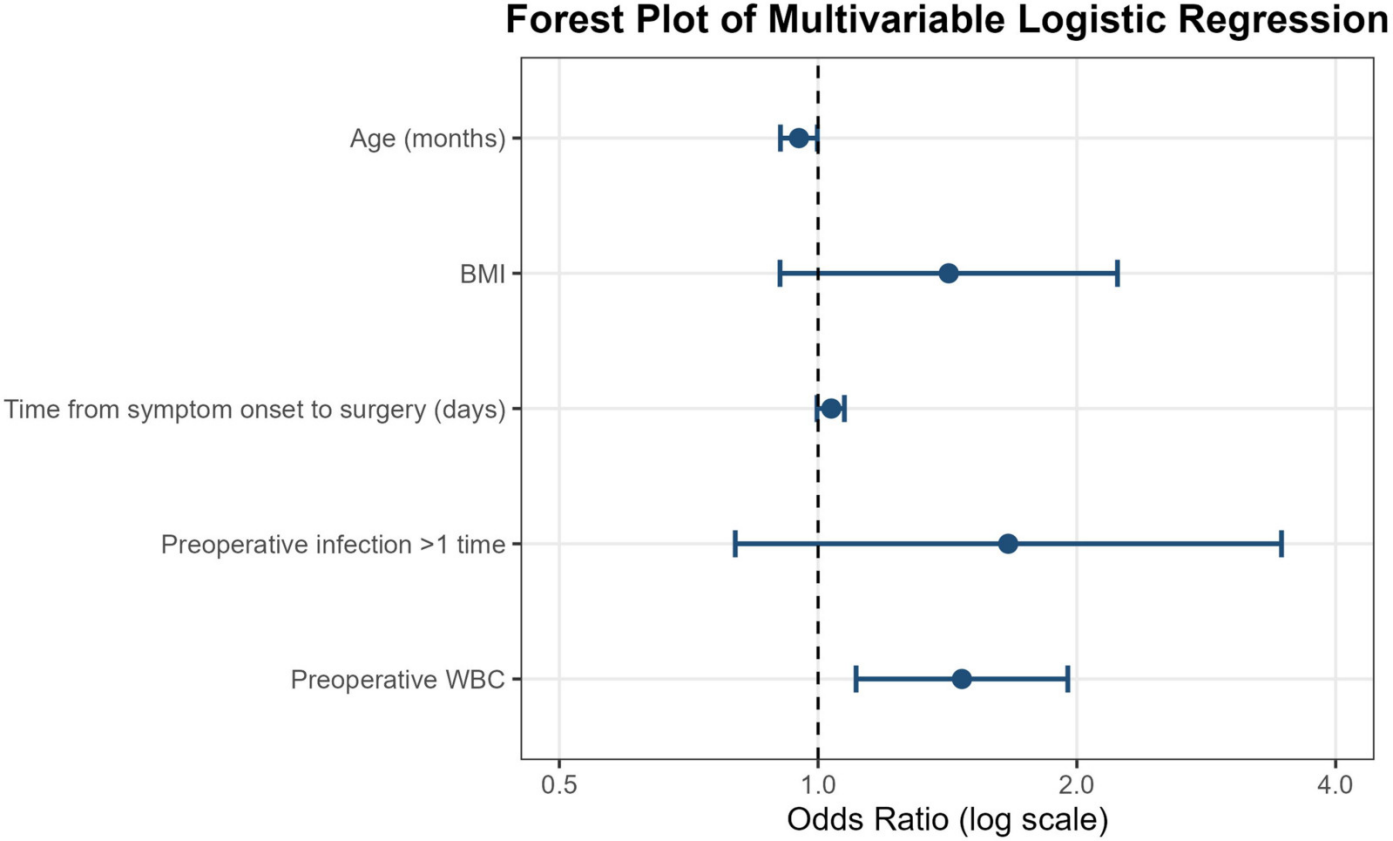
Forest plot of multivariate logistic regression analysis for transient vocal cord paralysis.

Variables with *P* < 0.10 in univariate analysis (age, BMI, interval from symptom onset to surgery, more than one preoperative infection episode, and preoperative WBC level) were entered into a multivariate logistic regression model using a stepwise approach. The results are shown in Table 3; Figure 3.

**Table 3:** Multivariate logistic regression analysis of transient vocal cord paralysis.

Variable	*β*	*SE*	*Wald χ 2*	*P*	*OR*	95% *CI*
Age	−0.051	0.025	4.160	0.041	0.950	0.904–0.998
BMI	0.350	0.230	2.315	0.128	1.419	0.903–2.230
Interval from symptom onset to surgery	0.035	0.018	3.780	0.052	1.036	0.997–1.073
Number of preoperative infection episodes (>1)	0.980	0.520	3.550	0.159	1.664	0.801–3.460
Preoperative WBC	0.385	0.145	7.050	0.008	1.470	1.107–1.952

Multivariate analysis demonstrated that higher age (OR = 0.950, 95% CI 0.904–0.998, *P* = 0.041) and preoperative WBC level (OR = 1.470, 95% CI 1.107–1.952, *P* = 0.008) remained independently associated with postoperative transient vocal cord paralysis. Younger age and elevated preoperative white blood cell count were independent risk factors. BMI, interval from symptom onset to surgery, and more than one preoperative infection episode did not show independent predictive value after adjustment for confounding factors (all *P* > 0.05).

### 3.4 Postoperative outcomes and long-term follow-up

All procedures were completed successfully. The median total operative time was 35 min (range, 10–65 min). Median intraoperative blood loss was 1 mL (range, 0–7 mL), and the median postoperative hospital stay was 11 days (range, 4–19 days). The median plasma radiofrequency ablation time was 60 s (range, 60–180 s). The internal opening of the pyriform sinus was clearly identified intraoperatively in all patients.

Postoperative transient vocal cord paralysis occurred in 11 patients (30.6%), all of whom developed hoarseness on postoperative day 1. The median duration of hoarseness was 8 days (range, 5–10 days), and complete recovery was observed in all cases. No permanent vocal cord paralysis or other serious perioperative complications occurred.

The median follow-up duration was 36 months (range, 3–72 months). During follow-up, recurrence occurred in only one patient (2.8%) at 2 months postoperatively. Recurrence was defined as the reappearance of clinical symptoms suggestive of cervical infection (such as neck swelling, pain, or fever), which is the primary indication for intervention in pyriform sinus fistula. Given that the disease typically manifests through recurrent infectious episodes, the absence of such symptoms during follow-up was considered indicative of successful fistula closure and was corroborated by laryngoscopic examination when symptoms arose. This patient was successfully treated with repeat endoscopic low-temperature plasma radiofrequency ablation and experienced no further recurrence. No recurrence was observed in the remaining 35 patients. The overall recurrence-free survival rate was 97.2% (35/36). All successfully treated patients remained free of cervical infection during follow-up, and laryngoscopic examination confirmed satisfactory closure of the internal opening of the pyriform sinus (Table 4).

**Table 4:** Postoperative outcomes and long-term follow-up results.

Variable	Data
Total operative time (min)	Median: 35 min (range: 10–65 min)
Intraoperative blood loss (mL)	Median: 1.5 mL (range: 0–7 mL)
Postoperative hospital stay (days)	Median: 11 days (range: 4–19 days)
Transient vocal cord paralysis	11 (30.6%)
Permanent vocal cord paralysis	0 (0.0%)
Other severe complications	0 (0.0%)
Recurrence	1 (2.8%)
Median follow-up time (months)	36 months (range: 3–72 months)
Recurrence-free survival rate	97.2% (35/36)

## 4 Discussion

The results of this study demonstrate that endoscopic low-temperature plasma radiofrequency ablation provides favorable long-term outcomes for pediatric PSF, with a low recurrence rate and good procedural reproducibility. These findings further support the central role of internal opening closure in the management of PSF. By interrupting the pathological communication between the pharyngeal cavity and cervical infectious foci, endoscopic treatment can mechanistically reduce repeated pathogen entry into the fistulous tract, thereby lowering the risk of recurrent infection and disease relapse (8). In the present study, the vast majority of patients remained free of cervical infection or fistula recurrence during follow-up, and laryngoscopic examinations confirmed satisfactory closure of the internal opening, supporting the effectiveness and durability of this treatment strategy.

Compared with traditional open fistula excision, endoscopic treatment offers clear safety advantages in pediatric patients. Previous studies have shown that although open surgery has curative potential, repeated infections or prior cervical incision and drainage often result in local fibrosis, which increases the difficulty of fistula identification and recurrent laryngeal nerve preservation. As a result, complication rates are significantly higher than those of endoscopic treatment, with an overall success rate of approximately 85% (2, 9–11). Consequently, an increasing number of studies advocate minimally invasive endoscopic strategies centered on internal opening closure as the first-line treatment for pediatric PSF, in order to maintain efficacy while reducing surgical risk (9, 10, 12).

Low-temperature plasma radiofrequency ablation generates plasma energy at relatively low temperatures, allowing effective destruction of the fistulous epithelium while minimizing thermal injury to surrounding nerves and vessels (13). Compared with other endoscopic techniques, this method provides better control over ablation depth and extent. Among alternative endoscopic options, CO₂ laser ablation offers precise cutting with minimal thermal spread but requires specialized equipment. Electrocautery is widely available but generates higher local temperatures, increasing the risk of collateral thermal injury. Chemical cauterization is less commonly used due to concerns about uncontrolled tissue damage. Therefore, low-temperature plasma ablation is particularly suitable for children, in whom anatomical structures are delicate and cervical space is limited (14, 15). Previous studies have also shown that even in cystic PSF, successful closure of the internal opening alone may lead to gradual shrinkage or resolution of the lesion (16–18), further emphasizing the pivotal role of internal opening management in disease control.

Regarding complications, postoperative transient vocal cord paralysis occurred at a certain frequency in this cohort but was uniformly temporary and fully reversible, with no cases of permanent nerve injury. Multivariate logistic regression analysis identified younger age and elevated preoperative white blood cell count as independent risk factors for transient vocal cord paralysis. These findings suggest that anatomical immaturity and preoperative inflammatory status play important roles in postoperative neural function changes.

Anatomically, younger children have thinner mucosa and looser submucosal tissue at the base of the pyriform sinus, and the recurrent laryngeal nerve and its branches run more superficially in this region. These features may increase susceptibility to indirect thermal conduction or local edema during ablation (14, 19, 20). In addition, pronounced preoperative inflammation may lead to tissue edema and increased release of inflammatory mediators, which can lower the tolerance of neural tissue to mechanical or thermal stress and thereby increase the risk of transient neural dysfunction (5, 21).

Regarding the differential predictive value of inflammatory markers, our analysis found that preoperative white blood cell (WBC) count, but not C-reactive protein (CRP) or procalcitonin (PCT), was independently associated with transient vocal cord paralysis. This difference may be attributable to several factors. First, WBC is a broad indicator of systemic inflammatory response, reflecting both acute and subclinical infection burden as well as overall immune activation. In contrast, CRP and PCT are more specific to acute bacterial infection and may normalize faster after preoperative antibiotic therapy, potentially reducing their sensitivity at the time of surgery. Second, the local inflammatory milieu around the fistula tract—often characterized by tissue edema and inflammatory cell infiltration—does not always correlate with systemic elevations in CRP or PCT, particularly in localized or partially treated infections. Therefore, WBC may represent a more stable and comprehensive marker of the underlying inflammatory status that influences neural tissue susceptibility to thermal or mechanical stress during ablation.

Strategies to mitigate the risk of transient vocal cord paralysis, particularly in younger children, should be considered. Based on our findings and technical experience, we recommend: (1) meticulous control of ablation energy and duration, with consideration for reducing the standard ablation depth (e.g., to 0.4 cm) in children under 2 years of age, provided that adequate mucosal discoloration is achieved; (2) continuous irrigation of the surgical field with normal saline during ablation to dissipate heat and reduce thermal conduction to adjacent neural structures; (3) employing a layered and circumferential ablation technique under high-definition visualization to precisely confine the treatment area; and (4) individualized timing of surgery, ensuring optimal control of local inflammation before intervention. Intraoperative neuromonitoring, though not used in this series, could be explored in high-risk cases as an adjunct for nerve localization and protection.

Beyond these intraoperative technical measures, the timing of surgery is another critical aspect of risk management. Traditional practice generally recommends delaying surgery until acute inflammation has fully resolved to reduce the risk of complications (8). However, recent studies suggest that, with strict patient selection and standardized technique, endoscopic low-temperature plasma radiofrequency ablation performed during the infectious phase in combination with abscess incision and drainage can achieve satisfactory outcomes without increasing the risk of permanent nerve injury (13, 22). In the present study, some patients underwent surgery shortly after infection control and achieved favorable results, indicating a certain degree of flexibility in surgical timing. Nevertheless, in clinical practice, individualized assessment based on patient age, inflammatory severity, and local anatomical conditions remains essential. Strict control of ablation range and energy parameters is critical to further reduce the risk of complications (10, 14).

This study has several limitations. First, it was a single-center retrospective study with a relatively small sample size, which may limit the generalizability of the findings and poses inherent constraints on the multivariate regression analysis. The limited cohort (*n* = 36) affects the statistical power of the model, increases the risk of overfitting, and reduces the precision of odds ratio estimates. To enhance the robustness of our analysis, we performed quality control checks including stepwise variable selection (entry *P* < 0.10), collinearity assessment, and reporting of both univariate and multivariate results with confidence intervals. Second, assessment of postoperative transient vocal cord paralysis was primarily based on clinical symptoms, laryngoscopic examination, and follow-up observations. Laryngeal electromyography was not routinely performed, which may have led to underestimation of mild or subclinical nerve dysfunction. Although the median follow-up duration was 36 months (range up to 72 months), and recurrence typically occurs within two years, multicenter prospective studies with larger cohorts are still needed to validate these risk factors and to explore more individualized surgical and neuroprotective strategies.

## 5 Conclusions

In conclusion, endoscopic low-temperature plasma radiofrequency ablation is a safe, effective, and repeatable minimally invasive treatment for pediatric PSF, with favorable long-term outcomes and a low recurrence rate. Although postoperative transient vocal cord paralysis is relatively common, it is usually reversible. Younger patients and those with more pronounced preoperative inflammatory responses are at higher risk for this complication. These findings highlight the importance of enhanced perioperative risk assessment in such patients, along with strict control of ablation extent and energy parameters and reinforced nerve protection strategies. Standardized surgical techniques combined with individualized treatment planning may further improve outcomes and reduce complication rates.

## Data Availability

The original contributions presented in the study are included in the article/Supplementary Material, further inquiries can be directed to the corresponding author.
